# Cost-effectiveness of chemotherapy for advanced and recurrent cervical cancer: a systematic review

**DOI:** 10.3389/frhs.2025.1616223

**Published:** 2025-07-28

**Authors:** Katsuaki Inami

**Affiliations:** Department of Obstetrics and Gynecology, Fujinomiya City General Hospital, Fujinomiya, Japan

**Keywords:** cervical cancer, cost-effectiveness, chemotherapy, immunotherapy, quality-adjusted life year, ICER, advanced cervical cancer, recurrent cervical cancer

## Abstract

**Introduction:**

Advanced and recurrent cervical cancer often requires palliative chemotherapy and is associated with poor prognosis. Recently, various systemic therapies—including cytotoxic drugs, anti-angiogenic agents, and immune checkpoint inhibitors—have been evaluated for their cost-effectiveness.

**Methods:**

We conducted a systematic review of English language-based research publications reporting incremental cost-effectiveness ratios (ICERs) for chemotherapy-based treatments in advanced or recurrent cervical cancer. Literature was retrieved from PubMed, Scopus, and Web of Science without date restrictions and screened based on predefined eligibility criteria. A total of 10 studies were included.

**Results:**

Traditional first-line platinum-based doublet chemotherapy (e.g., cisplatin plus paclitaxel) was consistently found to be cost-effective, with ICERs well below common willingness-to-pay (WTP) thresholds. The addition of bevacizumab improved survival but increased costs, yielding borderline or unfavorable ICERs (e.g., $155,000/QALY in the U.S.). Immunotherapy agents such as pembrolizumab and cadonilimab offered clinical benefits but often exceeded WTP thresholds, particularly in low- and middle-income settings. Cemiplimab had an ICER of $111,000/QALY as a second-line treatment, near the upper U.S. WTP threshold, while agents like tisotumab vedotin were not economically viable at current prices. Cost-effectiveness varied across regions depending on pricing, healthcare systems, and local WTP thresholds.

**Discussion:**

Although newer agents provide incremental survival benefits, their high costs often outweigh QALY gains. Policymakers and clinicians should consider the economic impact of adopting such therapies and prioritize value-based strategies, including price negotiations, biosimilar use, and biomarker-guided patient selection. Future research should promote evidence-based pricing and access models to support sustainable cancer care worldwide.

## Introduction

Cervical cancer remains a significant health burden worldwide, especially in advanced stages where cure is rarely achievable. In 2020, there were approximately 604,000 new cases of cervical cancer and 342,000 related deaths globally (1). A substantial proportion of these cases present as advanced disease or relapse after initial therapy. Advanced or recurrent cervical carcinoma (defined here as disease not amenable to curative surgery or radiotherapy) carries a poor prognosis, with 5-year survival under 20% in metastatic cases (1). Systemic chemotherapy is the mainstay of palliative treatment for these patients, and over the past two decades, various combinations of cytotoxic drugs—and more recently targeted therapies and immunotherapies—have been introduced to improve survival. For example, the addition of anti-angiogenic therapy (bevacizumab) to platinum-doublet chemotherapy showed a survival benefit in a landmark trial, and immune checkpoint inhibitors (like pembrolizumab) have demonstrated improved outcomes in PD-L1 positive cervical cancer in combination with chemotherapy. However, these novel therapies come at substantially higher costs. Given limited healthcare resources, it is crucial to assess whether the benefits of new treatments justify their costs. Cost-effectiveness analyses provide a structured economic evaluation by computing metrics such as the incremental cost-effectiveness ratio (ICER), which represents the additional cost per additional health outcome gained (often per QALY gained) when comparing a new intervention to standard care. An intervention is often considered “cost-effective” if the ICER falls below a willing-to-pay (WTP) threshold (commonly $50,000–$150,000 per QALY in the United States, or country-specific thresholds such as £20,000–£30,000 per QALY in the UK or three times GDP per capita in some health economic frameworks).

Several cost-effectiveness analyses have been conducted for advanced cervical cancer treatments. These studies vary by healthcare system perspective (e.g., U.S. payer, UK National Health Service, Chinese healthcare system), by the specific regimens compared, and by methodological assumptions. Early economic studies focused on cytotoxic chemotherapy combinations: for instance, whether adding a drug like paclitaxel or topotecan to cisplatin provides sufficient survival benefit to justify the increased cost. More recent analyses have evaluated adding targeted agents (bevacizumab) or immunotherapies (pembrolizumab, cemiplimab, cadonilimab) to chemotherapy, as well as newer agents for second-line therapy such as antibody-drug conjugates. To inform clinicians, patients, and policymakers, it is important to synthesize the evidence from these cost-effectiveness studies. We therefore performed a systematic review of published literature on the cost-effectiveness of chemotherapy (broadly defined to include chemotherapy +/- newer agents) in advanced or recurrent cervical cancer. Our aim was to summarize the reported ICERs and conclusions of each study, and to compare these across different treatments and settings. This systematic review adheres to the Preferred Reporting Items for Systematic Reviews and Meta-Analyses (PRISMA) guidelines and follows the author guidelines of *Frontiers in Health Services*.

## Methods

### Literature search strategy

We systematically searched multiple databases for relevant studies. The primary databases used were PubMed, Scopus, and Web **of Science**. The search strategy combined terms related to cervical cancer, chemotherapy, cost-effectiveness, and ICER. Keywords included “cervical cancer,” “advanced cervical,” “recurrent cervical,” “chemotherapy,” “cost-effectiveness,” “cost utility,” “economic evaluation,” and “ICER.” The following Boolean search string was used for PubMed: (“cervical cancer”[Title/Abstract] OR “advanced cervical”[Title/Abstract] OR “recurrent cervical”[Title/Abstract]) AND (“chemotherapy”[Title/Abstract] OR “systemic therapy”[Title/Abstract]) AND (“cost-effectiveness”[Title/Abstract] OR “cost utility”[Title/Abstract] OR “economic evaluation”[Title/Abstract] OR “ICER”[Title/Abstract]). Equivalent adaptations were applied for Scopus and Web of Science. Full search strings are provided in Supplementary Table S1. We placed no restrictions on publication date, in order to capture both older and recent studies. The search was limited to English-language publications, as our focus was on peer-reviewed literature in English. We also manually screened the reference lists of key articles to identify any additional relevant studies not captured by the database search. The final literature search was conducted on April 20, 2025.

**Figure 1:**
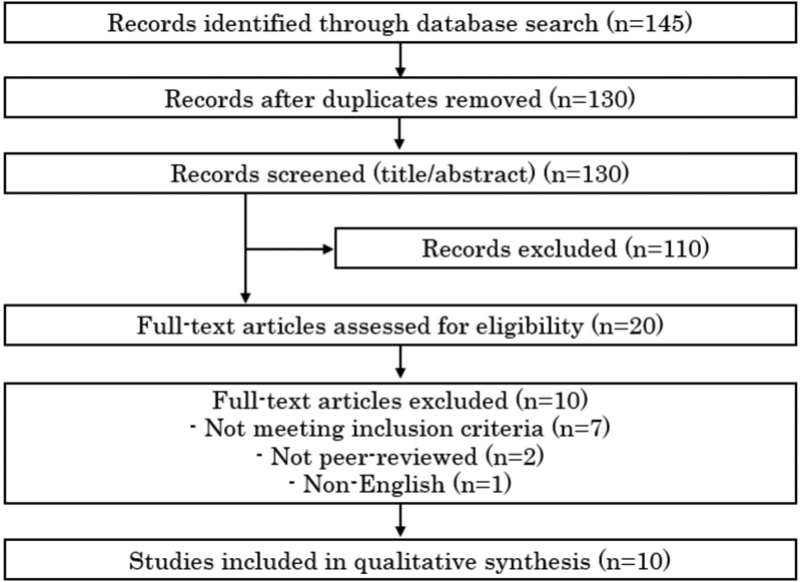
PRISMA flow diagram of study selection.

The initial search results underwent de-duplication to remove overlapping records across databases. Titles and abstracts were independently screened by two reviewers, including the author, in accordance with PRISMA recommendations. Disagreements were resolved through discussion. We obtained full-text articles for all studies that passed the title/abstract screening or for which eligibility was uncertain. Figure 1 presents a PRISMA flow diagram illustrating the study selection process.

### Inclusion and exclusion criteria

Studies were included in the review if they met the following criteria: (1) Population: patients with advanced, persistent, or recurrent cervical cancer (any study focusing on early-stage curative settings was excluded); (2) Intervention/Comparators: systemic therapy (chemotherapy alone or in combination with other agents such as targeted therapy or immunotherapy), with at least one comparison of chemotherapy-based regimens (e.g., comparing two different chemotherapy regimens, or chemotherapy with vs. without an added agent); (3) Outcomes: the study must report an incremental cost-effectiveness ratio (ICER)—for example, cost per QALY gained or cost per life-year gained—comparing the interventions; (4) Study type: full economic evaluations (cost-effectiveness or cost-utility analyses) published in peer-reviewed journals. We excluded conference abstracts, commentaries, and other secondary analyses unless they provided sufficient cost-effectiveness data. Non-English articles were excluded, as were studies that did not specifically evaluate advanced or recurrent disease (for instance, cost-effectiveness of cervical cancer screening or prevention were outside scope). We also avoided inclusion of any retracted articles by cross-checking each included study for retraction status and verifying that all data came from credible, published sources.

### Data extraction and synthesis

From each included study, we extracted key information: first author, publication year, country (setting) or perspective of analysis, the treatment strategies compared, and the main outcomes—particularly the ICERs (with currency and year of costing noted). Where available, we recorded whether the ICER was considered below that country's threshold (i.e., whether the authors concluded the intervention was “cost-effective” or not under typical thresholds). Any reported sensitivity analyses or noteworthy scenario analysis results (such as ICERs in subgroups) were also noted. Given the heterogeneity of interventions and economic models, a meta-analysis was not applicable; instead, we performed a qualitative synthesis of findings. We present a comparative table (Table 1) summarizing the ICERs from each study for ease of reference. All cost values are reported as per the original study (with currency noted; if needed, conversions or inflation adjustments are mentioned in the study results we cite). Where studies evaluated multiple comparisons, we extracted the ICER for the primary comparison of interest (e.g., new regimen vs. standard regimen).

**Table 1:** Summary of cost-effectiveness results (ICERs) from included studies evaluating chemotherapy regimens in advanced/recurrent cervical cancer.

Study (year, country)	Treatment comparison	ICER (incremental cost per QALY)	Conclusion
Geisler et al. (2012, USA) (2)	Cisplatin + Paclitaxel vs. Cisplatin	$13,654 per QALY (2011 USD)	Cost-effective (ICER <<$50k)
Geisler et al. (2012, USA) (2)	Cisplatin + Topotecan vs. Cisplatin	$152,327 per QALY (2011 USD)	Not cost-effective (high ICER)
Paton et al. (2010, UK) (3)	Cisplatin + Topotecan vs. Cisplatin (cisplatin-naive)	£10,928–£17,974 per QALY (2010 GBP)*	Cost-effective (≤£20k)
Paton et al. (2010, UK) (3)	Paclitaxel + Cisplatin vs. Topotecan + Cisplatin	£13,260 per QALY (2010 GBP)	Paclitaxel doublet preferred
Phippen et al. (2015, USA) (4)	Cisplatin/Paclitaxel + Bevacizumab vs. Chemo alone	$155,148 per QALY (2013 USD)	Not cost-effective at $100k threshold (borderline)
Barrington et al. (2022, USA) (5)	Chemo + Pembrolizumab vs. Chemo + Bevacizumab (1l)	$92,678 per QALY (2021 USD)	Cost-effective (∼$100k threshold)
Barrington et al. (2022, USA) (5)	Chemo + Pembro + Bevacizumab vs. Chemo + Bevacizumab	Dominated (more costly, less efficient)	Not cost-effective
Lin et al. (2023, China) (6)	Chemo + Pembro (+Bev) vs. Standard care (1l)	$114,276 per QALY (2021 USD)	Not cost-effective (>> threshold ∼$30k)
Ding et al. (2025, China) (10)	Chemo + Cadonilimab vs. Chemo (±Bev) (1l)	$75,945 per QALY (2022 USD)	Not cost-effective (threshold ∼$38k)
Liu et al. (2023, USA-perspective) (7)	Cemiplimab (2l) vs. Chemo in recurrent CC	$111,211 per QALY (2021 USD)	Marginally cost-effective (∼$150k WTP)
Huo et al. (2024, USA-perspective) (8)	Tisotumab vedotin (2l/3l) vs. Chemo	$839,108 per QALY (2023 USD)	Not cost-effective (ICER >> $150k)

*GBP* *=* *British Pound Sterling. (*): Paton 2010 reported different ICERs for subpopulations (cisplatin-naive vs. prior cisplatin).

We assessed the quality of the economic evaluations using basic criteria (e.g., clarity of perspective, inclusion of appropriate costs and outcomes, use of sensitivity analysis), drawing on the CHEERS checklist as a guiding framework. We assessed the quality of the economic evaluations using basic criteria (e.g., clarity of perspective, inclusion of appropriate costs and outcomes, use of sensitivity analysis), drawing on the CHEERS checklist as a guiding framework. While no formal risk of bias scoring tool was applied, we qualitatively considered key elements relevant to bias and methodological transparency, including study perspective, time horizon, discount rate, costing methodology, and funding source. These parameters were extracted and used to contextualize the results in the qualitative synthesis. However, given that all included studies were published in peer-reviewed journals, we assumed a baseline level of quality and focused primarily on results. Discrepancies in data extraction between reviewers were resolved by consensus.

### Compliance with PRISMA

This systematic review was conducted in compliance with PRISMA guidelines. A PRISMA flow chart (Figure 1) details the identification, screening, eligibility, and inclusion of studies. We have included all relevant PRISMA checklist elements in the reporting of methods, including search strategy, selection process, data collection, and synthesis. No protocol was registered for this review. A completed PRISMA 2020 checklist is provided as Supplementary Table S1.

### Use of AI-assisted writing tools

During the preparation of this manuscript, ChatGPT (OpenAI, GPT-4, accessed April 2025) was utilized to assist in language editing and structural suggestions. The authors have thoroughly reviewed and verified the accuracy and originality of all AI-assisted content to ensure compliance with ethical standards.

## Results

### Study selection and characteristics

Our search yielded 145 records after removing duplicates (Figure 1). After title and abstract screening, 20 articles were selected for full-text review. Of these, 10 studies met all inclusion criteria and were included in the qualitative synthesis [references (2–10)]. All included studies were full economic evaluations of chemotherapy regimens for advanced or recurrent cervical cancer, published in English peer-reviewed journals. Key characteristics and findings of these studies are summarized in Table 1. The studies spanned publication years 2010 through 2025, reflecting the evolution of available therapies in advanced cervical cancer. Four analyses were from a United States healthcare perspective (2, 4, 5, 8), including both academic and industry-supported evaluations. Two studies were from a United Kingdom/NHS perspective or related (one being a Health Technology Assessment report for NICE) (3). Four studies were from China's perspective (6, 7, 9, 10), reflecting cost-effectiveness in an environment of different drug pricing and lower WTP thresholds.

The treatments evaluated in these studies ranged from conventional chemotherapies (platinum doublets with paclitaxel or topotecan) to anti-angiogenic therapy (bevacizumab added to chemo) and immunotherapies (pembrolizumab, cemiplimab, cadonilimab) as part of first-line regimens, as well as a novel antibody-drug conjugate (tisotumab vedotin) in the second-line setting. All studies adopted a cost-per-QALY framework except one that also considered cost per life-year. The time horizon of analyses was typically long enough to capture lifetime costs and benefits (e.g., 5 years to lifetime). Below, we detail the findings by category of intervention.

### Cost-Effectiveness of first-line chemotherapy regimens

#### Platinum doublet chemotherapy vs. single-agent cisplatin

The combination of cisplatin and paclitaxel (a two-drug “doublet”) became a standard first-line chemotherapy for metastatic cervical cancer in the early 2000s, based on clinical trials showing improved response rates and a trend toward better survival over cisplatin alone. Geisler et al. (2012, USA) performed a cost-effectiveness analysis of this regimen, as well as cisplatin plus topotecan, using a decision model informed by Gynecologic Oncology Group (GOG) trials (2). They reported an ICER of $13,654 per QALY gained for cisplatin + paclitaxel compared to cisplatin alone (in 2011 USD). This indicates that adding paclitaxel had an acceptable cost per benefit gained, well below common thresholds (e.g., $50,000/QALY) and was considered cost-effective (2). By contrast, adding topotecan to cisplatin (another regimen tested in GOG-0179) yielded only a modest incremental survival benefit of ∼3 months but at higher cost; Geisler et al. calculated an ICER of $152,327 per QALY for cisplatin + topotecan vs. cisplatin. When all three regimens were compared, the topotecan combination was dominated (i.e., more costly and less effective than an alternative) in their model. These findings suggest that, from a U.S. perspective, cisplatin + paclitaxel is a cost-effective regimen, whereas cisplatin + topotecan is not justified by its high ICER (2). Similarly, a UK analysis by Paton et al. (2010) for NICE reported an ICER of £17,974/QALY for cisplatin + topotecan vs. cisplatin in cisplatin-naive patients. That analysis also compared multiple doublets and found paclitaxel + cisplatin to be more cost-effective than topotecan + cisplatin (ICER ∼£13,260/QALY for paclitaxel + cisplatin vs. topotecan + cisplatin), reinforcing that paclitaxel was the preferred addition. Overall, the evidence indicates that cisplatin/paclitaxel doublet chemotherapy offers good value and should remain the backbone against which newer therapies are evaluated in advanced cervical cancer, whereas adding **topotecan** was not cost-effective except perhaps in certain subsets (e.g., cisplatin-naive, where the ICER was lower) (3).

#### Addition of bevacizumab to chemotherapy

The anti-angiogenic antibody bevacizumab was shown in 2014 (GOG-240 trial) to improve median overall survival by ∼3.7 months when added to chemotherapy for advanced cervical cancer. However, bevacizumab is expensive, and its cost-effectiveness was in question. Phippen et al. (2015, USA) evaluated “Chemo + Bev” (typically cisplatin-paclitaxel plus bevacizumab) vs. chemotherapy alone, incorporating costs of drugs, administration, and management of side effects (4). They found that adding bevacizumab increased the total treatment cost from ∼$5,700 to ∼$53,800, and the ICER for chemo + bevacizumab was $155,000 per QALY gained. This ICER is above the commonly cited US WTP thresholds ($100k/QALY), meaning that bevacizumab was not clearly cost-effective at full price. Sensitivity analyses in that study showed the ICER would fall below $100,000/QALY if bevacizumab's cost was reduced by >37% or if a lower dose (7.5 mg/kg instead of 15 mg/kg) were used. The authors concluded that bevacizumab's “value” is marginal, approaching cost-effectiveness only with price discounts or dose optimizations. This analysis aligns with real-world considerations: many health systems have been cautious in adopting bevacizumab broadly due to its high cost relative to benefit. It is notable that $155k/QALY, while above traditional thresholds, is near the upper end of what some U.S. payers might consider; indeed, Phippen et al. commented that it “approaches common cost-effectiveness standards” (4). In other healthcare systems with stricter cost thresholds, bevacizumab would not be considered cost-effective. No included study from Europe specifically evaluated bevacizumab's cost-effectiveness, but by extrapolation, an ICER of £100k + per QALY would be far above NICE's limits. Thus, bevacizumab has only borderline economic justification and might require price reductions to be an efficient use of resources.

#### Addition of pembrolizumab (immunotherapy) to first-line chemotherapy

The advent of immunotherapy has introduced pembrolizumab (an anti-PD-1 antibody) as a new standard in 2021 for PD-L1 positive advanced cervical cancer in combination with chemotherapy. This combination significantly improves survival, but at very high drug cost (pembrolizumab can cost ∼$10,000 per cycle in the U.S.). Multiple cost-effectiveness studies have examined this scenario. Barrington et al. (2022, USA) performed a three-arm cost-effectiveness analysis comparing: chemotherapy + bevacizumab (CB) vs. chemotherapy + pembrolizumab (CP) vs. chemotherapy + pembrolizumab + bevacizumab (CPB), reflecting the possible regimens per the KEYNOTE-826 trial (5). They found that chemo + pembrolizumab (CP) had an ICER of $92,678 per QALY gained relative to chemo + bevacizumab (at a WTP threshold of $100k/QALY). By this analysis, CP was considered cost-effective (just under the threshold) in the U.S. context. In contrast, the triple combination CPB was dominated (more costly and not more effective than CP) in their base case, indicating that adding bevacizumab to pembrolizumab (with chemo) did not provide enough benefit to justify the huge additional cost. Barrington et al. also noted that if pembrolizumab's efficacy were slightly lower or if its cost were higher, the ICER of CP would quickly exceed $100k. In a subgroup of PD-L1 positive patients, the ICER for CP improved to ∼$63,670, reflecting better effectiveness in that subgroup. Overall, that study suggested pembrolizumab + chemo can be cost-effective by U.S. standards (especially in PD-L1 enriched populations), whereas adding bevacizumab to it is not cost-effective (5).

However, cost-effectiveness in other countries can differ. Lin et al. (2023, China) evaluated the first-line pembrolizumab + chemo + bevacizumab regimen in the Chinese healthcare system (6). In their partitioned survival model, they found that adding pembrolizumab (with chemotherapy ± bevacizumab as in the trial) yielded an ICER of US$114,276 per QALY gained (in 2021 USD). This vastly exceeded China's usual WTP threshold (around $30,000/QALY, roughly three times GDP per capita). Even after some “calibration” adjustments, the ICER remained ∼$52,766/QALY, still above the threshold in China (6). They concluded the pembrolizumab combination “may not be cost-effective” in China's system unless the drug price is substantially reduced. Similarly, a 2022 analysis by Shi Y et al. (2022, Gynecol Oncol, China/U.S. collaboration) found the ICER of pembrolizumab in the U.S. to be ∼$247,000/QALY when using a $100k threshold (thus not cost-effective), and even higher in lower-income settings (9). The differences between these findings highlight how results can vary with perspective: industry models and higher thresholds vs. more conservative assumptions or lower thresholds.

#### Cadonilimab plus chemotherapy

Cadonilimab is a bi-specific immunotherapy (anti-PD-1/CTLA-4) approved in China for cervical cancer. Ding et al. (2025, China) evaluated cadonilimab + chemo (± bevacizumab) vs. chemo (± bev) in the first-line setting based on the Phase III trial (COMPASSION-16) (10). They found an ICER of $75,945 per QALY for adding cadonilimab vs. standard therapy. At China's WTP threshold (∼$38,000/QALY), cadonilimab was not cost-effective, with a negative net monetary benefit and only a 0.7% probability of being cost-effective in probabilistic analysis. They noted the price of cadonilimab would need to drop by about 50% to reach the threshold. Thus, despite cadonilimab's clinical efficacy, its economic value in China appears unfavorable at current pricing (6). There are not yet published cost-effectiveness studies of cadonilimab from a Western perspective, but its cost (if similarly high) would likely pose a challenge there as well.

In summary, first-line immunotherapy combinations (pembrolizumab or cadonilimab with chemotherapy) markedly improve patient outcomes but often with ICERs above traditional cutoffs, especially in healthcare systems with lower expenditure thresholds. U.S. analyses using higher WTP benchmarks tend to label pembrolizumab + chemo as cost-effective (5), whereas analyses in China find it and cadonilimab not affordable under current pricing (6). Key drivers of these results are drug price, survival benefit magnitude, and threshold applied. Patient selection (e.g., treating only PD-L1 positive patients, who derive greater absolute benefit) can improve cost-effectiveness ratios modestly (5).

### Cost-effectiveness of second-line and subsequent therapies

Patients with cervical cancer who progress after first-line platinum-based chemotherapy have limited treatment options. Recently, new agents have been introduced in the second-line setting, including immunotherapy for those who did not receive it first-line, and targeted drugs like antibody-drug conjugates. The cost-effectiveness of these novel agents is an important consideration for their adoption.

#### Cemiplimab (Pd-1 inhibitor) in second-line

Cemiplimab was evaluated in the EMPOWER-Cervical 1 trial as a second-line therapy for recurrent/metastatic cervical cancer, demonstrating an overall survival benefit over chemotherapy. Liu et al. (2023, China, with U.S. perspective) assessed cemiplimab vs. chemotherapy in this setting from a U.S. payer perspective (7). Their Markov model over 20 years showed that cemiplimab provided an additional 0.597 QALYs compared to single-agent chemotherapy, at an increased cost of ∼$66,000, resulting in an ICER of $111,211 per QALY (2021 USD)​. This ICER is below the $150k threshold and thus they concluded cemiplimab is a cost-effective second-line option in the U.S (7). Notably, they found cemiplimab's cost to be the most influential factor; any increase in the drug price would raise the ICER above threshold. They also reported cemiplimab was more likely to be cost-effective in certain subgroups, such as patients with PD-L1 ≥ 1% tumors (which align with its indication). This suggests that in a U.S. context, cemiplimab's value is acceptable given its significant survival benefit and a price point slightly lower than pembrolizumab's. No similar analysis is available yet for other regions, but if applied to, say, a Chinese setting, the ICER would likely far exceed local thresholds (given the high cost and smaller budgets).

#### Tisotumab vedotin (antibody-drug conjugate) in second/third-line

Tisotumab vedotin is a novel antibody-drug conjugate approved for second-line treatment of recurrent cervical cancer (after chemotherapy). Huo et al. (2024, China, with U.S. perspective) evaluated tisotumab vedotin vs. “investigator's choice” chemotherapy (e.g., topotecan, vinorelbine, gemcitabine, etc.) in patients who had progressed on first-line chemo (8). The results were striking: tisotumab vedotin provided only +0.25 QALYs over chemo, at an additional cost of ∼$206,779, yielding an ICER of $839,108 per QALY (2023 USD). This is extremely high—well above any plausible threshold. The probability of cost-effectiveness at WTP $150k/QALY was essentially 0%. One-way sensitivity analysis showed that even large variations in parameters did not bring the ICER anywhere near acceptable levels; only massive reductions in the drug price would change the outcome (8). The authors concluded that tisotumab vedotin is not cost-effective at its current price for recurrent cervical cancer (8). Given this ICER, payers are unlikely to cover the drug broadly without discounts or unless further evidence shows a bigger benefit. This finding highlights that some cutting-edge therapies, despite being scientifically promising, may not be viable from a health economics standpoint unless costs are contained.

It should be noted that second-line chemotherapy itself (without these new agents) has limited efficacy, and historically there have been few cost-effectiveness analyses focusing on it. Many patients in this setting receive palliative chemotherapy (e.g., topotecan, or re-challenge with platinum) more for disease control or symptoms than for extending survival, making cost-effectiveness harder to quantify. The new targeted agents strive to improve survival where chemo offers little, but as shown, their cost per benefit can be exorbitant. Future analyses might consider the cost-effectiveness of sequential therapy (first-line immunotherapy and then second-line options) to guide optimal resource use across the treatment continuum.

### Summary of ICERs

Table 1 provides a comparative summary of the ICERs reported by each included study. The table lists the study reference (author, year, country), the treatments compared, and the ICER with currency. This allows quick visualization of which therapies are judged cost-effective or not in their respective contexts.

As shown in Table 1, earlier combinations like cisplatin-paclitaxel have very low ICERs (highly cost-effective), whereas adding new agents tends to raise ICERs substantially. Interventions with ICERs below typical thresholds in their context include cisplatin + paclitaxel (2, 3), cisplatin + topotecan in certain UK subgroups (3), and pembrolizumab + chemo in the U.S (5). Interventions generally not deemed cost-effective include cisplatin + topotecan (USA) (2), bevacizumab + chemo (USA, at full price) (4), pembrolizumab or cadonilimab combos in China (6), and tisotumab vedotin (USA) (8). Some, like cemiplimab (USA) (7), lie near the borderline. These results will be further interpreted in the discussion.

## Discussion

This systematic review compiled evidence from the literature on the cost-effectiveness of chemotherapy and related systemic therapies for advanced and recurrent cervical cancer. The findings illustrate a clear trend: older, established chemotherapy regimens (which are mostly generic drugs) offer good value, whereas newer therapies (bevacizumab, immunotherapies, targeted drugs) dramatically increase costs and often yield ICERs above commonly accepted thresholds. Below, we discuss the implications of these results, the factors influencing cost-effectiveness in this disease setting, and the limitations of available studies.

### Platinum doublets as cost-effective backbone

The combination of a platinum (cisplatin or carboplatin) with a taxane (paclitaxel) has been the standard palliative chemotherapy for advanced cervical cancer for nearly two decades. Our review confirms that this regimen is highly cost-effective when compared to older single-agent therapy. In both U.S. and U.K. analyses (2, 3), the ICER for adding paclitaxel to cisplatin was well below conventional WTP thresholds, essentially because paclitaxel (now a generic drug) is relatively inexpensive and provides a modest survival benefit with improved tumor response. The consistency of this finding across different healthcare systems reinforces that platinum-doublet chemotherapy should remain the fundamental first-line treatment from a value standpoint. In contrast, adding topotecan—which was a new drug in the 2000s—did not provide sufficient incremental benefit relative to its cost in most analyses (2, 3). GOG-179 had established cisplatin-topotecan as an efficacy winner over cisplatin alone, but the cost per QALY [∼$152k in the U.S (2)]. was unacceptably high and that regimen was largely superseded by the more cost-effective paclitaxel combination. This highlights that clinical significance does not always equate to economic viability, especially if a new drug is costly and the survival gain is small. Health technology assessors (like NICE in the U.K.) rightly scrutinized topotecan's value proposition, ultimately favoring the paclitaxel doublet which provided similar or better survival at lower cost (3).

### Cost-effectiveness of bevacizumab—price vs. benefit

The introduction of bevacizumab was a major development, improving median survival beyond 12 months for the first time in metastatic cervical cancer. Economically, however, bevacizumab is a classic case of high cost for moderate benefit. At its market price around 2014, the ICER for adding bevacizumab was approximately $155k/QALY in the U.S (4). This is above the traditionally cited $100k threshold, suggesting bevacizumab is *not* cost-effective, though some might argue it is within a “grey zone” (since some U.S. payers or analyses allow up to $150k/QALY). It is telling that sensitivity analyses showed a need for >37% price reduction to bring bevacizumab into the ∼$100k range (4). Many resource-constrained health systems likely decided against funding bevacizumab routinely for cervical cancer on this basis. Indeed, in some countries, access to bevacizumab for cervical cancer has been limited or delayed due to cost concerns. The findings from Phippen et al. (4) also underscore an important strategy: dose optimization. They posited that using a lower dose (7.5 mg/kg, as effective in ovarian cancer) could nearly halve the ICER. In practice, the cervical cancer trials used 15 mg/kg; if future trials or real-world practice consider a lower dose, the cost-effectiveness could improve. Another aspect is that bevacizumab's patent has since expired in many regions and biosimilars are available, which may lower its price. If the cost drops significantly (by 40%–50%), bevacizumab might become cost-effective in retrospect. Therefore, while bevacizumab was borderline at introduction, its economic profile may improve over time with biosimilars—a lesson for other biologics as well.

### Immunotherapy: high cost, targeted use

Immunotherapy has transformed the treatment of cervical cancer, with pembrolizumab emerging as a key agent. In U.S.-based analyses, pembrolizumab plus chemotherapy demonstrated ICERs between ∼$58,000 and $93,000 per QALY, generally considered cost-effective under U.S. thresholds (5). However, studies from China reported much higher ICERs, often exceeding $100,000 per QALY, well above national willingness-to-pay thresholds (6, 9). Subgroup analyses have suggested improved cost-effectiveness in patients with high PD-L1 expression (e.g., CPS ≥10). Interestingly, the addition or absence of bevacizumab did not substantially alter ICERs, as its cost impacts both arms similarly. These findings highlight that pembrolizumab's economic viability is context-dependent, influenced by drug pricing, threshold standards, and patient selection. Similar issues apply to cadonilimab, which—despite clinical benefit—was not cost-effective in Chinese analyses due to high acquisition costs (10). Interpreting ICERs requires contextual understanding of WTP thresholds, which vary widely across health systems. In high-income countries such as the United States, thresholds of $100,000–$150,000 per QALY are often applied, though not officially mandated. In contrast, the United Kingdom typically applies a lower threshold of £20,000–£30,000 per QALY via NICE guidelines. Middle-income countries like China often use thresholds based on GDP per capita, commonly ranging from 1 to 3 times GDP, resulting in markedly lower WTP benchmarks. This variation means that an ICER considered borderline in the U.S. (e.g., $111,000/QALY for cemiplimab) may be entirely unaffordable in other settings. For LMICs, WTP thresholds are often below $10,000/QALY, rendering most newer agents economically unviable without major price reductions or external funding mechanisms. Therefore, WTP assumptions must be interpreted carefully, and cost-effectiveness should not be extrapolated across regions without contextual analysis. Our synthesis incorporated author-reported WTP benchmarks, but critical interpretation was necessary to assess whether these aligned with national health economic norms.

### Second-line therapies: diminishing returns and high costs

Second-line treatments for cervical cancer often offer modest survival benefits in a refractory setting, yet come with substantial drug costs. For example, tisotumab vedotin was associated with an ICER of over $800,000 per QALY, far exceeding any acceptable threshold due to its limited survival benefit and high price (8). In contrast, cemiplimab showed a more favorable ICER of $111,000 per QALY in the U.S., though this still exceeds common $100k benchmarks and would be unaffordable in lower-income settings (7). These cases illustrate that high-cost therapies with incremental benefits are unlikely to be economically viable unless prices are significantly reduced or clinical efficacy improves.

### Cross-comparisons and health policy implications

It is instructive to compare interventions: For example, $155k/QALY for bevacizumab vs. $93k/QALY for pembrolizumab (in U.S.)—one might argue pembrolizumab provides more value for money relative to bevacizumab, especially given it can induce long-term remission in a subset of patients. Indeed, some U.S. value frameworks (like ICER in the US) did evaluate pembrolizumab; an early analysis (2021) found pembrolizumab would need a price reduction to be cost-effective at $100k/QALY, aligning with Barrington et al.'s findings. This has implications: companies might engage in value-based pricing or patient access schemes. In the UK, NICE initially did not recommend pembrolizumab for cervical cancer until a confidential discount was likely arranged to improve its cost-effectiveness profile. Thus, these analyses are not merely academic—they directly influence reimbursement decisions.

### Economic modeling considerations

Cost-effectiveness results can vary depending on the perspective and time horizon adopted. While most studies used a payer perspective, incorporating a societal view—including productivity impacts—may slightly improve ICERs. Similarly, lifetime time horizons can favor treatments like immunotherapy, which may yield long-term survivors. However, such extrapolations and differences in modeling approaches (e.g., partitioned survival vs. state-transition) introduce uncertainty. Therefore, results should be interpreted within the context of each model's assumptions.

### Limitations of the evidence

This review has several limitations, which are grouped into four thematic areas:

1. Methodological and Modeling Heterogeneity

The included studies varied substantially in their methodologies, including differences in modeling approaches, time horizons, discount rates, perspectives (payer vs. societal), and outcome measures (e.g., overall survival vs. QALYs). Drug prices also differed significantly across countries and over time. This heterogeneity complicates direct comparison of ICERs and makes meta-analytic synthesis inappropriate. Most studies did include QALY-based outcomes, which supports some degree of comparability. However, due to these differences, we opted for a structured qualitative synthesis rather than pooled ICER calculations, which would have been statistically misleading given the context-specific assumptions underlying each study.

Additionally, many studies were based solely on model-driven economic evaluations and did not incorporate real-world data. As such, the findings may not fully reflect actual clinical effectiveness, treatment patterns, or healthcare resource use. This limits the external validity of the results and highlights the importance of future research that integrates real-world evidence.

2. Generalizability and Geographic Representation

This review included only studies published in English, which may have introduced language bias. Consequently, cost-effectiveness analyses conducted in non-English-speaking regions—particularly in Latin America, sub-Saharan Africa, and parts of Asia—may have been unintentionally excluded. This could result in the underrepresentation of regions with a high burden of cervical cancer and may reduce the global applicability of the findings.

Although some studies from China were included, many other low- and middle-income countries (LMICs) such as India, Nigeria, and countries in Central America were not represented. This absence likely reflects broader publication trends, language limitations, and differences in national research priorities, rather than an intentional exclusion. Countries like Japan and India are highly relevant given their disease burden and evolving access to novel therapies, yet few eligible studies from these regions met our inclusion criteria.

3. Risk of Bias and Authorship Limitations

Some of the included studies were sponsored by pharmaceutical companies [e.g., Merck for pembrolizumab (5)], which may lead to favorable conclusions based on optimistic assumptions. In contrast, independently funded analyses may adopt more conservative estimates (5, 6). We included both types and highlighted such differences when appropriate to present a balanced perspective.

While we considered several quality elements aligned with the CHEERS checklist—such as clarity of perspective, cost inclusion, and sensitivity analysis—a formal quality scoring system was not applied. Furthermore, this review was conducted by a single author. Although predefined eligibility and extraction criteria were used, the lack of independent screening or data verification constitutes a methodological limitation that should be addressed in future updates through collaboration.

4. Timeliness of Cost Data and Treatment Evolution

As treatments evolve and drug prices change—due to biosimilar entry or national pricing negotiations—some ICERs reported in earlier analyses may no longer be reflective of current cost-effectiveness. For example, the 2015 ICER for bevacizumab may now be an overestimate due to recent price reductions. We attempted to flag where such changes could materially affect interpretation, but this dynamic landscape underscores the need for periodic re-evaluation of cost-effectiveness conclusions.

In addition, many studies did not account for complex treatment pathways such as combination sequencing or crossover between treatment arms, which are increasingly relevant in real-world oncology practice. These omissions may lead to underestimation or overestimation of true cost-effectiveness in clinical settings, especially for high-cost drugs like immunotherapies used beyond first-line.

### Research and policy outlook

Going forward, cost-effectiveness research in advanced cervical cancer should continue to incorporate real-world data, especially as longer-term survival data from immunotherapy become available. If, for example, 10%–20% of patients have durable 5-year survival on pembrolizumab, how do we value that? Additionally, combination approaches (e.g., immunotherapy plus another agent) though currently extremely costly [as CPB showed (5)], might be optimized or targeted to those who need both. Another area is biosimilars and generics: as patents expire, the landscape can change (e.g., a pembrolizumab biosimilar in the future could dramatically improve cost-effectiveness if priced lower). Health systems in resource-limited settings may consider strategies like compulsory licensing or price negotiations for these life-extending drugs to improve affordability.

For policymakers, this review highlights that improvements in survival must be weighed against quality of life and cost. In advanced cervical cancer, quality of life is a crucial component (as treatments can cause toxicity). Many analyses incorporated QALYs, which factor in quality of life decrements due to side effects. It is reassuring that even with these factored in, some interventions (like pembro + chemo) still produced a substantial QALY gain (5). It emphasizes that beyond the dollars, the *value* includes enabling patients to live longer and possibly with better quality of life. Nonetheless, the high ICERs of some therapies mean tough decisions in allocation of healthcare funds. Investing in prevention (HPV vaccination, screening) remains the most cost-effective approach to reducing cervical cancer burden in the long term (though that was outside our scope, it is worth noting). Meanwhile, for those patients who do develop advanced disease, we need to find ways to maximize their outcomes in a cost-conscious manner.

## Conclusion

In advanced and recurrent cervical cancer, systemic chemotherapy regimens display a wide range of cost-effectiveness profiles. Conventional platinum-based doublet chemotherapy is cost-effective and remains the recommended backbone therapy. The incorporation of bevacizumab improves survival at a relatively high cost, yielding ICERs that generally exceed traditional willingness-to-pay thresholds unless the drug cost is discounted. The addition of immune checkpoint inhibitors like pembrolizumab significantly extends survival in eligible patients and can be considered cost-effective in high-income settings that use high thresholds, but is not cost-effective in lower-income settings under current pricing. Newer agents for second-line treatment, such as cemiplimab, show borderline cost-effectiveness in the U.S., whereas tisotumab vedotin is far from cost-effective at its current price. Regional differences are pronounced: what is cost-effective in one country may not be in another due to variations in drug pricing and threshold standards.

To improve the cost-effectiveness of care for advanced cervical cancer, strategies could include price reductions for high-cost drugs, use of biosimilars, and patient selection to identify those most likely to benefit from expensive therapies. Managed access agreements or outcomes-based pricing could also be considered for very costly drugs (e.g., paying only if a patient responds). As new treatments emerge (including combination immunotherapies), concurrent economic evaluation should guide their adoption to ensure sustainability. Ultimately, the goal is to maximize both the quantity and quality of life for patients with advanced cervical cancer while judiciously utilizing healthcare resources. Ongoing research and dialogue between clinicians, health economists, payers, and industry will be essential to achieve equitable and cost-effective cancer care. This systematic review contributes uniquely to the current literature by synthesizing not only established therapies, but also emerging cost-effectiveness data for novel agents such as cadonilimab and tisotumab vedotin, which have not yet been comprehensively discussed in prior institutional reports or WHO analyses. By aggregating findings from diverse healthcare systems—including the United States, China, and the United Kingdom—this review also highlights how cost-effectiveness is context-dependent, shaped by local pricing, payer perspective, and WTP thresholds. Through this comparative perspective and focus on policy-relevant metrics, the study provides actionable insights for clinicians, policymakers, and payers navigating the evolving treatment landscape of advanced cervical cancer.

## Data Availability

The original contributions presented in the study are included in the article/Supplementary Material, further inquiries can be directed to the corresponding author.
